# TFProtBert: Detection of Transcription Factors Binding to Methylated DNA Using ProtBert Latent Space Representation

**DOI:** 10.3390/ijms26094234

**Published:** 2025-04-29

**Authors:** Saima Gaffar, Kil To Chong, Hilal Tayara

**Affiliations:** 1 Department of Electronics and Information Engineering, Jeonbuk National University, Jeonju 54896, Republic of Korea; saimagaffar@jbnu.ac.kr; 2 Advances Electronics and Information Research Centre, Jeonbuk National University, Jeonju 54896, Republic of Korea; 3 School of International Engineering and Science, Jeonbuk National University, Jeonju 54896, Republic of Korea

**Keywords:** transcription factors, methylated deoxyribonucleic acid, machine learning, non-methylated deoxyribonucleic acid, protein language model, bidirectional encoder representations from transformers

## Abstract

Transcription factors (TFs) are fundamental regulators of gene expression and perform diverse functions in cellular processes. The management of 3-dimensional (3D) genome conformation and gene expression relies primarily on TFs. TFs are crucial regulators of gene expression, performing various roles in biological processes. They attract transcriptional machinery to the enhancers or promoters of specific genes, thereby activating or inhibiting transcription. Identifying these TFs is a significant step towards understanding cellular gene expression mechanisms. Due to the time-consuming and labor-intensive nature of experimental methods, the development of computational models is essential. In this work, we introduced a two-layer prediction framework based on a support vector machine (SVM) using the latent space representation of a protein language model, ProtBert. The first layer of the method reliably predicts and identifies transcription factors (TFs), and in the second layer, the proposed method predicts and identifies transcription factors that prefer binding to methylated deoxyribonucleic acid (TFPMs). In addition, we also tested the proposed method on an imbalanced database. In detecting TFs and TFPMs, the proposed model consistently outperformed state-of-the-art approaches, as demonstrated by performance comparisons via empirical cross-validation analysis and independent tests.

## 1. Introduction

Transcription factors (TFs) are proteins pivotal in cell-fate decisions involved in gene regulation and expression. These TFs are found throughout the genome, and the number of TFs present tends to correlate with the size of the genome [1]. Larger genomes typically contain a greater abundance of TFs on average. It is estimated that around 10% of genes in the human genome encode TFs, making them one of the most abundant families of proteins in humans. Transcription factors are intimately associated with the process of transcribing DNA into RNA. They serve as crucial regulators, interacting with gene promoters to control expression. By recruiting numerous RNA polymerases to the core promoter region, TFs can either hasten or slow down the transcription of genetic information from DNA to RNA. The diverse functions of different cells sharing the same genetic material can be attributed to TFs, which influence cell differentiation by modulating the expression of particular genes. Moreover, TFs regulate multiple genes at various cell stages, but their activity is constrained by the specific DNA binding sites. Mutations found in transcription factors (TFs) have been identified as contributors to numerous diseases [2]. TFs serve as critical switches that regulate the precise timing, location, and extent of gene expression, ensuring accurate control over gene activation or repression. Therefore, accurately identifying transcription factors (TFs) is not only a fundamental challenge but also an opportunity for therapeutic advancements in drug discovery and development. Accurately identifying true TFs allows researchers to focus on proteins that directly regulate gene expression, especially genes involved in disease pathways. TFs have three primary functional regions, namely binding to DNA, regulation of transcriptional activity, and signal transduction [3,4]. These unique segments are essential for binding to the specified gene’s promoter [5]. In the early stages, DNA methylation primarily targeted cytosines, particularly within cytosine–guanine dinucleotides (CpGs), as a means to regulate the affinity of transcription factors (TFs) [6,7,8]. Initially, DNA methylation was recognized as a suppressive mark on transcription, wherein its presence at CpG-rich gene promoters, known as CpG islands, hindered TF binding, thereby leading to gene inactivation. However, with advancements in high-throughput sequencing and the development of novel profiling techniques for TF binding, along with the introduction of third-generation sequencing methods, a vast array of DNA modification sequences have been sequenced and identified. This has been made possible through techniques such as bisulfite sequencing, PacBio single-molecule real-time sequencing, and Oxford Nanopore sequencing [9]. Therefore, recent studies have verified that certain transcription factors lacking methyl-CpG binding domains (MBDs) can still engage with methylated DNA [8,10,11]. Understanding the mechanism behind the interaction between transcription factors and methylated DNA holds considerable importance in comprehending methylation-related biological diversity [12,13,14]. Traditional methods, such as physical or chemical assays (referred to as wet experiments), offer a primary means of TF identification. These wet experiments encompass various techniques like SELEX-based methods [15], MITOMI [16], and ChIP-based assays [17]. Many known TFs have been uncovered through these wet experiments and cataloged in public databases [18,19,20,21]. However, while wet experiments have revealed a considerable number of TFs, they have consumed substantial time and financial resources and are insufficient to comprehensively identify all TFs across various tissues and species worldwide. Advancements in artificial intelligence offer a promising avenue for addressing this challenge. By leveraging computational models trained on known TFs, it is now possible to predict and identify new, previously unknown TFs. These predicted TFs can then be validated through wet experiments. Computational methods significantly reduce the pool of potential TF candidates that need to be screened via wet experiments, thus saving significant time and resources. As such, computational methods are increasingly viewed as complementary to wet experiments, collectively accelerating the discovery and exploration of TFs.

To our understanding, Liu et al., in 2020 [22], introduced the initial computational approach, TFPred, for distinguishing transcription factors (TFs) from non-TFs. They used hand-crafted compositional encoding types like composition/transition/distribution (CTD), amino acid composition (AAC), and di-peptide composition (DPC). Subsequently, the support vector machine (SVM) algorithm was employed to ascertain whether the sequence corresponds to a transcription factor (TF). Nevertheless, the characteristics outlined in the aforementioned models fail to retain the original sequence information that records the position of amino acids. It is important to remember that changing one amino acid or the locations of many amino acids can have a big impact on the protein’s functional characteristics and spatial structure. Nevertheless, these alterations have little impact on conventional characteristics, which results in insufficient transcription factor (TF) descriptions. In 2022, Q.H. Nguyen [23] proposed a technique, PSSM + CNN, that employs the position-specific scoring matrix (PSSM) to encode protein sequences for transcription factor (TF) classification. Subsequently, a deep convolutional neural network (CNN) was utilized to identify TFs. However, the accuracy of PSSM greatly depends on the quality of sequence alignment, making it susceptible to errors. Additionally, factors such as database size, diversity, and sequence length can impact the creation of PSSM profiles, potentially leading to inaccuracies in the extracted features and reducing their reliability. Zheng et al. [24] proposed a capsule network-based architecture, Capsule_TF, inspired by the work of Sabour et al. [25], to identify TFs. Li et al., in 2022 [26], introduced a novel approach, Li_RNN, distinct from TFPred, for discriminating between transcription factors (TFs) and non-TFs. Rather than devising complex features, Li et al., in their model, Li_RNN, directly utilized the sequence as input, dividing it into basic units of three amino acid residues, and applied long short-term memory (LSTM) to capture semantic distinctions between TFs and non-TFs. Due to the restricted data availability, capsule networks, RNNs, and LSTMs are prone to overfitting. Given that these networks require adjustment of multiple hyperparameters, identifying an optimal combination can be challenging.

**Figure 1:**
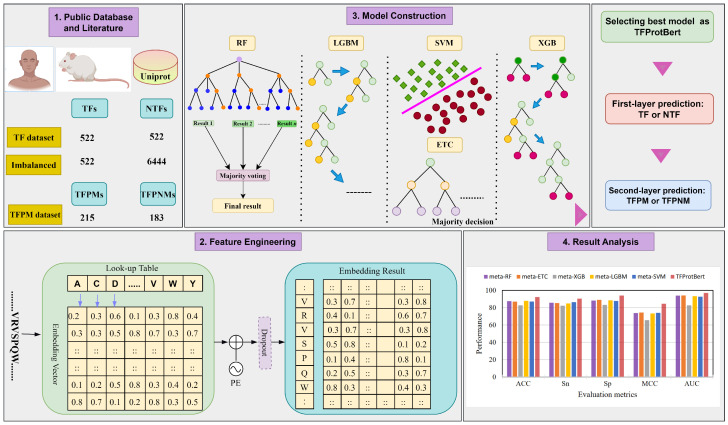
Framework of the proposed architecture of TFProtBert.

The rise of transformers and large language models (LLMs) has prompted the creation of novel deep learning frameworks tailored for scrutinizing protein sequences. Amino acid sequences are likened to linguistic constructs, enabling LLMs to adeptly grasp both straightforward and nuanced relationships within textual data. This resurgence of interest in bioinformatics stems from the recognition that, akin to languages, protein sequences exhibit intricate interactions among amino acids. Leveraging LLMs and transformers, scientists are now equipped to utilize advanced language modeling methods to delve into the functions of amino acids in shaping protein characteristics. Taking advantage of transformers to address the aforementioned drawbacks of previous models, particularly the challenge of dealing with extremely limited datasets, we present a method that uses a pre-trained model, ProtBert, for embedding, and then, using transfer learning, we are able to showcase exceptional performance in classifying transcription factors (TFs) and, subsequently, in predicting their ability to bind to methylated DNA. The architecture of the proposed method is shown in Figure 1.

## 2. Results

### 2.1. Performance Evaluation of the Baseline Models on Different Feature Encodings

**Figure 2:**
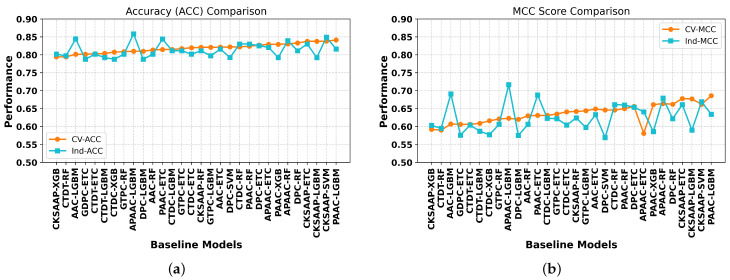
Using accuracy (ACC) and MCC score criteria, the performance of the top 30 baseline models is assessed. Figure (**a**,**b**) display the cross-validation and independent accuracy and MCC score, respectively, on the benchmark dataset.

To establish the best model for transcription factor identification, we carried out an extensive analysis of 45 different baseline models. We used independent testing as well as cross-validation on the given datasets to provide a robust evaluation. Supplementary Tables S1 and S2 contain the comprehensive findings for each dataset. An extensive examination of the top 30 baseline models is shown in Figure 2, with an emphasis on the models’ prediction accuracy and robustness in identifying transcription factors as demonstrated by accuracy and MCC scores. The PAAC and CKSAAP descriptors were found to be the most effective feature descriptors against LGBM and SVM classifiers, with CV ACC and MCC scores of 84.13% and 0.686 and 83.81% and 0.662, respectively. The top three ML classifiers were LGBM, SVM, and ETC, achieving CV accuracies of 84.13%, 83.81%, and 83.77%, respectively. It was discovered that the GDPC, APAAC, CTDT, GTPC, and CTDC feature encodings were underperforming with respect to the SVM classifier, achieving accuracies in the range of 72.17–76.89%.

The independent testing revealed that the best three models were APAAC-LGBM, CKSAAP-SVM, and AAC-LGBM, with ACC and MCC values of 85.84% and 0.717, 84.91% and 0.669, and 84.44% and 0.691, respectively. The performance of the baseline models on the independent set showed variations in terms of accuracies and MCC values with cross-validation values, as can be seen from Figure 2. For our prediction challenge, single-feature approaches showed limited generalizability and the performance was not sufficient. In order to get around this, we used a stacked ensemble learning technique, based on the probabilistic feature vectors, which produced a model that is more stable and broadly applicable, as evidenced by independent testing and cross-validation.

### 2.2. Performance Evaluation of the Meta-Models Using Probabilistic Feature Vectors

**Figure 3:**
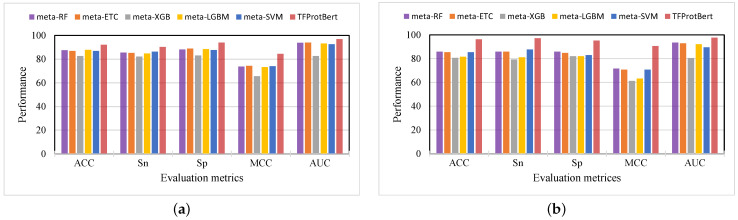
The performance comparison between the five meta-models trained on the 45-D probabilistic feature vector and TFProtBert trained on the 1024-D embedding vector. (**a**) The cross-validation performance on the benchmark training dataset. (**b**) The independent dataset performance.

After combining the outputs of the 45 baseline models, a 45-dimensional probabilistic feature vector was constructed. Five machine learning classifiers used this input to construct their corresponding meta-models. Using the 45-dimensional probabilistic feature vector, our investigation showed that all meta-modeling techniques performed in a generally consistent manner. Each meta-model’s performance is broken down in Figure 3.

Using cross-validation to evaluate the five meta-models, we discovered that the MCC scores ranged from 0.656 to 0.743 and the accuracies varied from 82.69 % to 87.85 %. Of all the meta-models, meta-LGBM performed best, with an MCC score of 0.733 and an accuracy of 87.85%, and meta-XGB was the least performing model, with an ACC of 82.69% and an MCC value of 0.656.

The meta-RF classifier performed best on the independent dataset, with an MCC score of 0.716 and an accuracy of 85.84%. With an MCC score of 0.707 and an accuracy of 85.37%, meta-ETC and meta-SVM both fared well. On the other hand, out of all the meta-models, meta-XGB and meta-LGBM performed the worst on the independent set, with accuracies of 80.66% and 81.60%, and MCC scores of 0.613 and 0.632, respectively. Accuracy ranged from 79.24% to 87.73% and MCC scores from 0.613 to 0.716 on the independent set. Even while the best meta-model, meta-LGBM, performs better than most single-feature-based models, there is still a great deal of space for enhancement. To further improve the outcomes, transformer embeddings were applied.

### 2.3. Performance Evaluation of the ML Classifiers with TFProtBert Using 1024-D Vector

We trained the five conventional machine learning classifiers using the ProtBert embedding vector to evaluate their cross-validation and independent set performances. From Table 1, the 5-fold cross-validation outcomes of ML models trained on the 1024-D vector vary from 88.05% to 92.15% for accuracy, and 0.763 to 0.845 for MCC, thereby confirming the robustness of the models. Among our models, the SVM classifier, named TFProtBert, demonstrates the highest performance, achieving a cross-validation accuracy of 92.15%, an MCC of 0.845, a sensitivity of 92.15%, a specificity of 93.99%, and an AUC of 96.93%. The TFProtBert method, achieves better performance than the 45 baseline models as well as the five meta-models, as can be seen from Figure 3.

For the independent dataset, the ML models surpass the 45 baseline models and the meta-models across all metrics, with results ranging from 87.73% to 96.22% for accuracy and 0.756 to 0.906 for MCC. The TFProtBert demonstrates the highest performance among all five models trained on the 1024-D embedding vector, with an accuracy of 96.22%, an MCC of 0.906, a sensitivity of 97.16%, a specificity of 95.28%, and an AUC of 97.59%, as depicted in Table 1.

### 2.4. Performance Evaluation Between TFProtBert and the Existing Methods

To demonstrate the effectiveness of the proposed approach, TFProtBert, its performance was assessed using the independent dataset in comparison with other established approaches, including the TFPred [22], PSSM + CNN [23], Capsule_TF [24], and Li_RNN [26] methods. It is important to note that the performance results of the approved procedures were taken straight out of the original papers that accompanied each technique. The proposed method, TFProtBert, achieved an accuracy of 96.22% and an MCC value of 0.906, surpassing the existing methods on all metrics, as can be seen from Table 2. Because TFProtBert uses the 1024-D embedding vector as an input, it has better discriminative capabilities than the existing techniques. The Capsule_TF method is the best existing method with an accuracy of 88.20%, an MCC value of 0.765, and an AUC value of 92.54%, which are 8.02%, 14.1%, and 5.05%, respectively, less than the TFProtBert values.

The ACC, Sn, Sp, MCC, and AUC score achieved by the TFProtBert method are 8.02–13.2%, 5.65–16.97%, 9.43–11.32%, 14.1–24.5%, and 1.63–6.43% higher than the existing methods. The potency of the TFProtBert model over the current approaches is demonstrated by its performance on both benchmark and independent datasets. The TFProtBert has a better discriminative ability to distinguish TFs from NTFs than the state-of-the-art methods by utilizing the ProtBert latent space representation.

### 2.5. Construction of the Second-Layer Model to Predict TFPMs and Its Comparison with the Existing Predictors

Applying the same process from the first-layer model, TFProtBert, to predict TFs, we used the TFPM dataset to develop the second prediction model regarding the prediction of the TFPMs. We used the ProtBert transformer with 30 embedding layers and 16 attention heads to generate a 1024-D-long embedding vector. This 1024-D vector was used as an input to the LGBM classifier to construct the second-layer prediction model. The independent performances of the second-layer prediction model and the existing method are mentioned in Table 3. We used the online web server of the Li_RNN method https://bioinfor.nefu.edu.cn/TFPM/ (accessed on 2 October 2024) to generate the results. As can be seen from Table 3, the LGBM classifier achieves an accuracy of 55.56% and an MCC score of 0.077. The accuracy of the Li_RNN model is meager 26.42%, and the MCC value is −0.483. For the sake of simplicity, the LGBM classifier was also named TFProtBert. These results clearly show that the performance of our model is much better than the existing method.

### 2.6. Performance of TFProtBert on Imbalanced Dataset

The generalization ability and robustness of the TFProtBert method were tested using the imbalanced dataset in predicting TFs from NTFs. The same methodology as for the TF dataset, we used the ProtBert method to extract a 1024-D-long embedding for the 416 positive and 6444 negative sequences. This 1024-D vector was used as input for the five machine learning classifiers, and the performance of all classifiers on the imbalanced dataset is mentioned in Table 4. From Table 4, SVM (named as TFProtBert) is the best classifier among all in terms of cross-validation recall and MCC values of 84.50% and 0.881. On the independent dataset, the SVM classifier again outperformed the other classifiers, achieving a recall value of 91.50% and an MCC score of 0.908. The recall and MCC scores of the SVM classifier, on the independent set, were 12.21–33.01% and 11.8–18.21% higher than the other four models. The discrepancy between sensitivity and specificity is slightly big, and this stark contrast is a direct result of the extreme imbalance within the dataset.

### 2.7. Two-Dimensional Feature Set Representation

**Figure 4:**
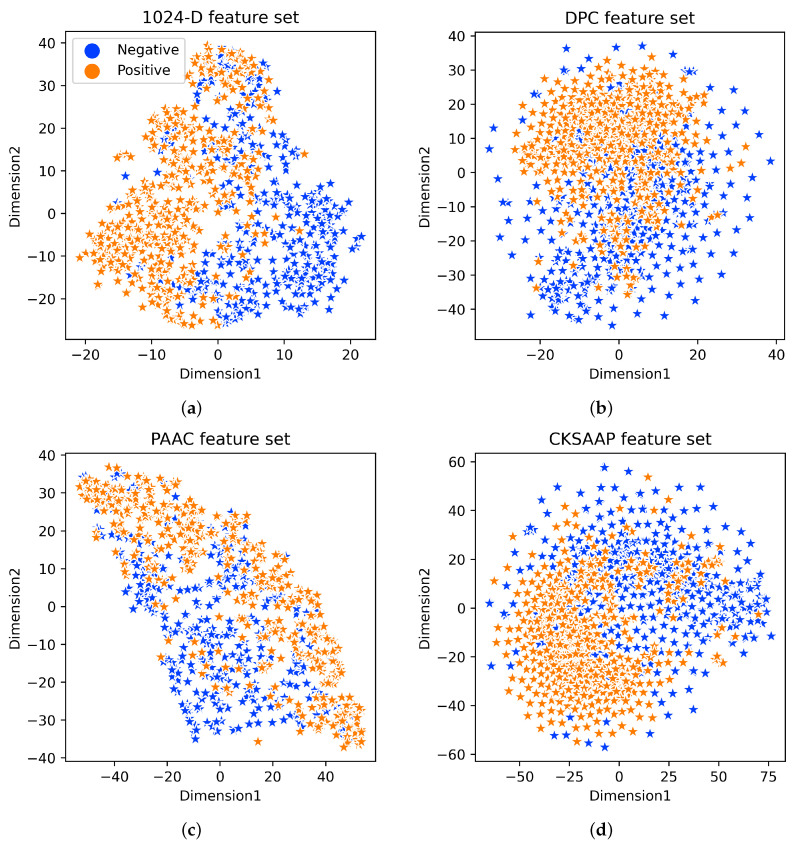
The t-distributed stochastic neighbor embedding (t-SNE) distribution of positive and negative samples on the benchmark dataset (**a**): 1024-D vector, (**b**) DPC, (**c**) PAAC, and (**d**) CKSAAP.

To provide a more thorough demonstration of the capabilities of TFProtBert, the data point arrangement in the 2D feature space of the TF dataset was represented using the t-distributed stochastic neighbor embedding (t-SNE) technique [27]. This technique is commonly employed in the bioinformatics field [28,29]. The 1024-D feature embedding vector and the top three single-feature descriptors (PAAC, DPC, and CKSAAP) were included in this representation. Figure 4 illustrates how light yellow and light blue dots, respectively, were used to symbolize TFs and NTFs in this depiction. The positive and negative samples were overlapping, as can be seen from Figure 4b–d, and this clearly demonstrates the limited capacity of the top three individual descriptors to discriminate TFs from NTFs in the feature space. The real characteristics in the single feature space may be insufficient to distinguish between TFs and NTFs. Nevertheless, in the feature space of the 1024-D feature vector, we found two separate clusters for positive and negative samples with a little overlap, as shown in Figure 4a. This suggests that the underlying patterns between positive and negative data are shown by the 1024-D feature vector. The study presented here clarifies how 1024-D representation from the top three descriptors may predict TFs with a high degree of discrimination and distinguish between samples that are positive and negative in the feature space.

## 3. Materials and Methods

### 3.1. Data Collection

The benchmark training and testing datasets were obtained from Liu et al.’s work [22]. The original dataset comprised 601 human and 129 mouse transcription factors (TFs) with a preference for methylated DNA [6,30], along with 286 TFs showing a preference for non-methylated DNA [3]. Liu et al. (TF dataset 2020) [22] refined the dataset through several steps to enhance its quality. Initially, sequences containing invalid characters such as “X”, “B”, and “Z” were excluded. Subsequently, redundancy among sequences was reduced using the CD-HIT clustering tool [31,32], with the threshold set at 0.25 to ensure the sequence identity did not exceed this value. Additionally, sequences with fewer than 50 amino acids were removed. Following these steps, 522 TFs were retained as positive samples. For negative samples, Liu et al. [22] randomly selected an equal number of non-TFs from the UniProt database while ensuring they met specific criteria: reviewed proteins, evidence at the protein level, full-length proteins with over 50 amino acid residues, and proteins lacking DNA-binding TF activities. The proteins seen in the CD-HIT have less than 25% sequence identity. The TF dataset consists of 522 positive and 522 negative samples.

For the first-layer model, the TF dataset was split between training and independent sets in the ratio of 80:20. The benchmark training set consists of 416 positive sequences and an equal number of negative sequences, whereas the independent set carries 106 positive sequences and 106 negative sequences.

For the second-layer prediction, identifying TFs preferring to be bound to methylated DNAs (TFPM dataset), the training set included 146 TFs not connected to methylation DNAs (negative class) and 146 TFs bound to methylated DNAs (positive class), and the independent set consisted of 37 TFs not connected to methylated DNAs (negative class) and 69 TFs attached to methylated DNAs (positive class).

Apart from the two datasets, we also used an imbalanced dataset to test the robustness and the generalization ability of the proposed model. To ensure the reliability of our model, we introduced a new set of protein sequences into the negative dataset. The new sequences were downloaded from the UniProt database (Release 2024_02) by using the keywords “NOT transcription activity AND evidence at the protein level AND reviewed: yes AND length between 50 and 500”. In total, 8887 protein sequences were included, and subsequently, we employed the CD-HIT [31] program at c = 0.4 to process our dataset, resulting in a final count of 6444 protein sequences. This dataset comprises proteins that meet several stringent criteria to ensure high quality and reliability. Specifically, the proteins included do not participate in transcription activity, thus excluding any proteins involved in the synthesis of RNA from a DNA template. Additionally, these proteins have lengths ranging between 50 and 500 amino acids, providing a focused range of sequence sizes. Each protein has direct experimental evidence confirming its existence, ensuring that its presence is validated through robust experimental methods such as mass spectrometry or X-ray crystallography. Furthermore, all entries have been reviewed and validated by experts, guaranteeing that the dataset consists of thoroughly vetted and high-quality protein records. The details of the three datasets are mentioned in the Table 5.

### 3.2. Feature Extraction

Machine learning prediction approaches rely on numerical values or feature vectors as their input. For suitable computational analysis, various methods, such as one-hot encoding or encoding based on physicochemical properties, are employed initially to convert peptide or protein sequences into numerical vectors. We used nine popular sequence-based feature encoding types apart from ProtBert embedding values in the prediction of TFs and for comparison as well. These nine encoding types were AAC, PAAC, APAAC, CKSAAP, CTDT, CTDC, GTPC, GDPC, and DPC and can be defined as:

(i) Amino Acid Composition (AAC): AAC [33] is a feature vector of length 20, representing the frequency of each amino acid’s occurrence within a given peptide sequence. The mathematical expression for AAC is as follows:
  (1) $$x\left(m\right)=\frac{L_{m}}{L},\;m\;\;\epsilon \left\{A,C,D,\ldots ,Y\right\}$$
  Here, *L* represents the total length of the sequence, while $L_{m}$ denotes frequency of the occurrence of the amino acid type *m*.
(ii) Pseudo-amino acid composition (PAAC): PAAC [34] translates protein or peptide sequences into numerical characteristics, capturing both the intrinsic attributes of each amino acid and its significant position within the sequence. The PAAC can be described as:
  (2) $$S=[S_{1},S_{2},\ldots ,S_{20+1},\ldots ,S_{20+\lambda }]$$
  with
  (3) $$S_{z}=\frac{x_{z}}{\sum_{j=1}^{20}x_{j}+w\sum_{k=1}^{\lambda }\theta_{k}},\;\left(1\leq z\leq 20\right)$$
  (4) $$S_{z}=\frac{w\theta_{z-20}}{\sum_{j=1}^{20}x_{j}+w\sum_{k=1}^{\lambda }\theta_{k}},\;\left(21\leq z\leq 20+\lambda \right)$$
  (5) $$\theta_{\lambda }=\frac{1}{L-\lambda }\sum_{m=1}^{L-\lambda }\Theta \left(S\left(R_{m}\right),S\left(R_{m+\lambda }\right)\right),\lambda <L$$
  where $x_{z}$ is the number of times the amino acid *z* occurs, *w* is the weight, set at 0.5, *L* is the sequence length, and $\theta_{\lambda }$ represents sequence-correlated factors. $\Theta (S\left(R_{m}\right),S\left(R_{m+\lambda }\right)$ implies a correlation function, and the value of λ is set at 3, making the PAAC feature a 23-D-long vector.
(iii) Amphiphilic pseudo-amino acid composition (APAAC): APAAC [35] takes into consideration the amphiphilic properties of amino acids, allowing for the depiction of protein sequences in terms of their hydrophobic and hydrophilic features. The computation process for APAAC is outlined as follows:
  (6) $$S=[S_{1},S_{2},\ldots ,S_{20},S_{20+1},\ldots ,S_{20+\lambda },\ldots ,S_{20+2\lambda }]$$
  with
  (7) $$S_{z}=\frac{x_{z}}{\sum_{j=1}^{20}x_{j}+w\sum_{k=1}^{2\lambda }\tau_{k}},\;\left(1\leq z\leq 20\right)$$
  (8) $$S_{z}=\frac{w\tau_{z}}{\sum_{j=1}^{20}x_{j}+w\sum_{k=1}^{2\lambda }\tau_{k}},\;\left(21\leq z\leq 20+2\lambda \right)$$
  in this context, *w* denotes the weight, which is assigned a value of 0.5, $x_{z}$ is the normalized occurrence of the amino acid *z*, and $\tau_{k}$ implies the sequence-order factor. These factors related to sequence order can be denoted as:
  (9) $$\tau_{2\lambda }=\frac{1}{L-\lambda }\sum_{k=1}^{L-\lambda }H_{k,k+\lambda }^{2}$$
  (10) $$\tau_{2\lambda -1}=\frac{1}{L-\lambda }\sum_{k=1}^{L-\lambda }H_{k,k+\lambda }^{1}$$
  the value λ is established as 3, resulting in an APAAC encoding of a 26-dimensional vector, while *L* is the peptide sequence.
(iv) Composition of *k*-spaced amino acid pairs (CKSAAP): The method described in [36] for CKSAAP feature encoding is utilized to analyze protein or peptide sequences. To find the frequency of *k*-spaced amino acid pairs in a protein or peptide sequence, this method involves a series of computations. Since the parameter *k*, in this case, fluctuates from 0 to 5, we concentrated on descriptors, especially for *k* = 5, which produced a 2400-dimensional feature vector for CKSAAP.
(v) Composition, transition, distribution, and triplet (CTDT): CTDT [37] is a feature representation approach employed in bioinformatics to analyze protein or peptide sequences. Its objective is to encompass diverse elements of the sequence, such as amino acid composition, shifts between distinct amino acid types, their arrangement patterns, and triplet arrangements.
(vi) Composition, transition, distribution, and composition (CTDC): CTDC [37] encoding aims to capture various facets of the sequence, including the frequency of amino acids, transitions between different types of amino acids, their spatial distribution, and other aspects related to amino acid composition.
(vii) Di-peptide composition (DPC): DPC [33] gives 400 descriptors based on the frequency of the two amino acids in a given sequence. It can be defined as:
  (11) $$t\left(m,n\right)=\frac{Z_{mn}}{Z-1},\;\;\;m,n\in \left[A,C,D,\ldots ,Y\right]$$
  where $Z_{mn}$ is the number of the di-peptides of type *m* and *n*, and *Z* denotes the sequence length.
(viii) Grouped di-peptide composition (GDPC): GDPC [38] is a special variation of the TDPC descriptor of 125 descriptors.
  (12) $$t\left(m,n\right)=\frac{Z_{mn}}{Z-1},\;\;\;m,n\in \left[g_{1},g_{2},g_{3},g_{4},g_{5}\right]$$
  where $Z_{mn}$ represents a di-peptide of amino acid types *m* and *n*, and *Z* represents the total length of the sequence. $g_{i}$ represents the five different classes.
(ix) Grouped tri-peptide composition (GTPC): GTPC [38] is a special variation of the TPC descriptor of 125 descriptors.
  (13) $$t\left(m,n,o\right)=\frac{Z_{mno}}{Z-2},\;\;\;m,n,o\in \left[g_{1},g_{2},g_{3},g_{4},g_{5}\right]$$
  where $Z_{mno}$ represents a tri-peptide of amino acid types *m*, *n*, and *o*, and *Z* represents the total length of the sequence. $g_{i}$ represents the five different classes.

### 3.3. Conventional Machine Learning-Based Classifiers

Numerous applications of ML, CNN, and GNN (convolutional and graph neural networks) may be found in image analysis [39,40,41,42,43], chem-informatics [44,45,46], and bioinformatics [28,47,48,49,50]. Nine encoding methods were employed in this study: AAC, PAAC, APAAC, CKSAAP, CTDT, CTDC, DPC, GTPD, and GDPC. The iLearn Python code [36] was utilized to produce the feature encodings previously described. Five traditional machine learning classifiers were utilized to construct the baseline models: support vector machines (SVMs), random forests (RFs), extra tree classifiers (ETCs), light gradient boosting machines (LGBMs), and extra gradient boosting (XGB). Numerous bioinformatics disciplines frequently utilize these classifiers [51,52,53]. Nine different descriptors, along with 5 conventional ML models, yielded 45 baseline models. We used the Optuna hyperparameter [54] to fine-tune each baseline model across several feature sets for the optimized framework. The hyperparameter range details are summarized in Supplementary Table S3.

### 3.4. Construction of Meta-Models

This study used nine different descriptors: AAC, PAAC, APAAC, DPC, CKSAAP, CTDT, CTDC, GTPC, and GDPC, alongside five distinct classifiers, RF, XGB, LGBM, ETC, and SVM, for constructing prediction models. We used the Optuna framework [54] to optimize each baseline model against every descriptor during cross-validation (CV) analysis. We assessed and scrutinized the impact of these nine descriptors and machine learning classifiers in identifying TFs and TFPMs. The combination of the five classifiers and nine feature encodings resulted in 45 prediction models. The output from the 45 baseline models was concatenated to generate a 45-dimensional probabilistic feature vector. In this context, the output (probability score) is interpreted as a probabilistic score (*PS*) ranging from 0 to 1. For a specific protein, denoted as *S*, its feature vector is formed by consolidating the probability scores from all 45 single-feature-based models. This process can be expressed as follows:

(14) $$vec\left(S\right)={\left[PS_{X(1)},PS_{X(2)},PS_{X(3)},\ldots \ldots ,PS_{X(45)}\right]}^{T}$$

where $PS_{X(m)}$ is the *m*th predicted probability from the *m*th baseline model. The vec(S) is a 45-D-long probabilistic feature vector for protein *S*. The five conventional ML classifiers utilized this 45-D vector as an input to create their corresponding meta-models. The performance of these five meta-models was evaluated against the proposed model TFProtBert.

### 3.5. Construction of TFProtBert

ProtBert is a special deep learning model based on the transformer architecture, inspired by the BERT (Bidirectional Encoder Representations from Transformers) model. ProtBert is specially designed for bioinformatics and computational biology tasks, particularly those involving peptide or protein sequences. The ProtBert was trained on the UniRef and Big Fantastic Data (BFD) databases. The ProtBert model treats each amino acid in a given sequence as a special token as input and outputs a representation useful for protein/peptide-related tasks. ProtBert, being a variant of BERT, uses the same configurations as BERT’s larger models to capture the complexities of peptide or protein sequences effectively. The number of layers with pre-trained weights used as the embedding layer and attention heads in ProtBert is 30 and 16, respectively. The output from the final embedding layer is a 1024-D-long vector. The 1024-D embedding is the input to the machine learning models (SVM and LGBM) to develop robust and efficient predictive classifiers for the two-layer problem, and it is termed TFProtBert.

For the first-layer prediction, the TFProtBert method has to identify and predict TFs and NTFs and is based on an SVM model. In the second-layer prediction, the TFProtBert has to predict TFPMs and TFPNMs, based on the LGBM model, provided that the method has predicted TF in the first-layer prediction. The TFProtBert is a two-layer prediction method. The TFProtBert will also be tested and tried on an imbalanced dataset.

### 3.6. Performance Evaluation

To evaluate the predictive model’s performance, it is required to verify the predictions’ robustness and generalizability [55,56,57,58,59,60]. To assess the abilities of the proposed method, we employed the following metrics: accuracy (ACC), sensitivity (Sn), specificity (Sp), the Matthews correlation coefficient (MCC), precision (Pr), F1 Score (F1), and the area under the curve (AUC). Except for the MCC metric, all other metrics are given in percentage values. These metrics can be defined as:

(15) $$\begin{array}{c} Accuracy\left(ACC\right)=\frac{Tp+Tn}{Tp+Fn+Tn+Fp} \end{array}$$

(16) $$\begin{array}{c} Sensitivity\left(Sn\right)=\frac{Tp}{Tp+Fn} \end{array}$$

(17) $$\begin{array}{c} Specificity\left(Sp\right)=\frac{Tn}{Tn+Fp} \end{array}$$

(18) $$\begin{array}{c} Precision\left(Pr\right)=\frac{Tp}{Tp+Fp} \end{array}$$

(19) $$\begin{array}{c} F_{1}Score\left(F1\right)=\frac{2*Precision*Sensitivity}{Precision+Sensitivity} \end{array}$$

(20) $$\begin{array}{c} MCC=\frac{\left(Tp\times Tn)-(Fp\times Fn\right)}{\sqrt{\left(Tp+Fp)\times (Tp+Fn)\times (Tn+Fp)\times (Tn+Fn\right)}} \end{array}$$

where Tp denotes true positive, Tn denotes true negative, Fp denotes false positive, and Fn denotes false negative.

## 4. Conclusions

Transcription factors are essential proteins that regulate gene transcription by binding to specific DNA sequences. They exert significant control over the timing and location of gene expression, thereby influencing crucial biological processes such as development, differentiation, and responses to environmental cues. Several computational predictors tailored to transcription factors have been devised to facilitate the development of drugs targeting these transcription factors. Introducing a novel approach to identify transcription factors, we utilize ProtBert, a transformer technique commonly applied to protein sequences, for embedding. These embeddings are then fed into five machine learning models, namely RF, ETC, XGB, LGBM, and SVM. The proposed model TFProtBert, an SVM based model, outperforms state-of-the-art models, achieving the highest accuracy of 96.22%, MCC of 0.906, sensitivity of 97.16, specificity of 95.28, and AUC of 97.59 on the main (balanced) independent dataset in predicting TFs from NTFs by utilizing the ProtBert latent space representation. The same is true for the second layer, in which the proposed method distinguishes between TFPMs and TFNPMs and achieves better results than the existing models on the independent dataset. The results achieved by the proposed model, TFProtBert, on both layers are a testament to the fact that the proposed model has achieved the intended goals comprehensively.

To assess the resilience of our model, we introduced an imbalanced negative dataset. The outcomes obtained from this new imbalanced dataset surpass those of the other models, demonstrating recall, MCC, and Pr values of 91.50%, 0.908, and 86.47%, respectively. However, it is important to note a notable difference between the sensitivity and specificity, which can be attributed to the highly imbalanced nature of the dataset.

Because the current study uses a small dataset of transcription factors, the predictor’s resilience when used on a varied transcription factor collection may be limited. Retraining the predictor with an updated dataset that includes new transcription factors from the literature will be crucial to improving its robustness. The predictor’s efficacy is further restricted by using a negative dataset consisting of randomly selected proteins. A negative dataset should ideally include non-transcription factors that have been confirmed by experimentation. But in their absence, random proteins with comparable physicochemical characteristics to non-transcription factors would be a better substitute. Furthermore, using novel feature representations may considerably improve transcription factor identification. These suggestions ought to be taken into account for upcoming attempts to construct models. All the datasets, source code, and models are made available on GitHub at https://github.com/Mir-Saima/TF (accessed on 22 April 2025).

## Figures and Tables

**Table 1:** The performance evaluation of ML classifiers and TFProtBert trained on the TF dataset using the 1024-D embedding vector.

Models	Benchmark Dataset					Independent Dataset				
	**ACC**	**Sn**	**Sp**	**MCC**	**AUC**	**ACC**	**Sn**	**Sp**	**MCC**	**AUC**
RF	90.94	89.58	92.06	0.818	96.13	91.50	90.56	92.45	0.830	95.28
ETC	90.58	88.61	91.82	0.807	96.21	91.98	91.50	92.45	0.839	95.86
XGB	88.05	87.16	88.94	0.763	94.88	87.73	90.56	84.90	0.755	94.97
LGBM	91.43	90.07	90.08	0.810	96.17	93.86	95.28	92.45	0.877	97.18
TFProtBert	92.15	90.31	93.99	0.845	96.93	96.22	97.16	95.28	0.906	97.59

**Table 2:** The performance comparison between TFProtBert and the existing methods on the independent dataset.

Method	ACC	Sn	Sp	MCC	AUC
TFPred	83.02	80.19	85.85	0.661	91.16
Li_RNN	86.63	88.68	83.96	0.727	91.30
PSSM+CNN	87.26	90.56	83.96	0.746	95.96
Capsule_TF	88.20	91.51	84.96	0.765	92.54
TFProtBert	96.22	97.16	95.28	0.906	97.59

The results for the existing methods were obtained directly from the original papers.

**Table 3:** The performance comparison with the existing method for distinguishing TFPM from TFPNM based on the independent dataset.

**Method**	**ACC**	**Sn**	**Sp**	**MCC**	**AUC**
Li_RNN	26.42	30.43	18.93	−0.483	—
TFProtBert	55.66	59.42	48.64	0.077	53.72

**Table 4:** The performance evaluation of ML models on the new imbalanced benchmark and independent datasets using 1024-D embedding.

Models	Imbalanced Benchmark Dataset							Independent Dataset						
	**ACC**	**Sn**	**Sp**	**MCC**	**AUC**	**Pr**	**F1**	**ACC**	**Sn**	**Sp**	**MCC**	**AUC**	**Pr**	**F1**
RF	97.09	66.58	99.53	0.768	97.35	92.98	72.61	97.27	70.75	99.45	0.790	96.79	91.49	79.70
ETC	97.21	74.09	99.06	0.786	97.43	92.08	70.31	96.55	58.49	99.68	0.726	97.51	93.91	72.04
XGB	97.21	74.09	99.06	0.786	97.43	86.55	79.77	96.70	68.86	98.99	0.747	99.44	84.87	76.07
LGBM	97.09	75.54	98.81	0.780	96.50	83.90	79.31	96.91	79.24	98.37	0.779	96.84	80.00	79.64
TFProtBert	98.41	84.50	99.53	0.881	97.92	91.61	78.40	98.70	91.50	99.30	0.908	97.94	86.47	82.18

**Table 5:** Details of the three datasets used in this study.

Dataset	Training Set		Independent Set	
	**Pos**	**Neg**	**Pos**	**Neg**
TF dataset	416	416	106	106
TFPM dataset	146	146	69	37
Imbalanced dataset	416	5155	106	1289

## Data Availability

All the datasets, source code, and models are made available on GitHub at https://github.com/Mir-Saima/TF (accessed on 22 April 2025).

## Supplementary Materials

The following supporting information can be downloaded at: https://www.mdpi.com/article/10.3390/ijms26094234/s1.
