# Supplementary material for: TFProtBert: Detection of Transcription Factors Binding to Methylated DNA Using ProtBert Latent Space Representation
Source: Int J Mol Sci. 2025 Apr 29;26(9):4234. doi: 10.3390/ijms26094234 (PMC12071566; doi:10.3390/ijms26094234)
Supplement: Supplementary file 1 [file ijms-26-04234-s001.zip › ijms-3576773-supplementary.pdf]

TFProtBert: Detection of transcription factors binding to methylated DNA using ProtBert latent space representation

Saima Gaffar[1], Kil To Chong[1,2,*], and Hilal Tayara[3,*]

[1]Department of Electronics and Information Engineering, Jeonbuk National University, Jeonju, 54896, South Korea
[2]Advances Electronics and Information Research Centre, Jeonbuk National University, Jeonju, 54896, South Korea
[3]School of International Engineering and Science, Jeonbuk National University, Jeonju, 54896, South Korea

Table S1: Search range of hyperparameters used during the optimization of the machine learning classifiers using Optuna framework

**Model**	**Parameters**	**Tested Values**
Random Forest	n_estimators	100-1000
	max_depth	Jan-60
	min_samples_split	Feb-20
	max_leaf_nodes	2-1000
	n_trails	200
XGB	n_estimators	50-1000
	max_depth	1-400
	learning_rate	0.001-10.0
	min_child_weight	Jan-50
	subsample	0.01-1.0
	colsample_bytree	0.01-1.0
	reg_alpha	1e-8 to 1.0
	reg_lambda	1e-8 to 1.0
	gamma	1e-8 to 10.0
	n_trails	200
ETC	n_estimators	100-2000
	max_depth	Oct-90
	max_leaf_nodes	15-100
	criterion	[gini, entropy]
	n_trails	200
LGBM	n_estimators	100-1000
	learning_rate	0.0001-10
	num_leaves	2-100
	max_depth	1-100
	min_child_samples	1-100
	subsample	0.7-1.0
	colsample_bytree	0.7-1.0
	reg_alpha	0.0-1.0
	reg_lambda	0.0-10.0
	n_trails	200
SVM	kernel	[linear, poly, rbf]
	svc_c	1e0-1e2
	n_trails	200

Table S2: The performance evaluation of the baseline models on the benchmark TF training set

Encoding	Model	ACC	SN	SP	MCC	AUC
**AAC**	RF	81.37	84.13	78.60	0.630	91.24
	ETC	82.13	84.13	80.52	0.649	92.01
	XGB	78.72	81.97	75.48	0.579	85.33
	LGBM	80.16	82.93	77.4	0.607	90.54
	SVM	79.19	83.69	75.04	0.599	86.77
**APAAC**	RF	83.05	81.25	84.85	0.664	92.01
	ETC	82.93	81.73	84.13	0.581	90.13
	XGB	78.96	79.08	78.84	0.581	84.25
	LGBM	81.00	80.52	81.49	0.623	88.75
	SVM	75.35	78.86	80.01	0.571	84.02
**PAAC**	RF	82.39	81.25	83.41	0.650	91.79
	ETC	81.49	79.80	83.17	0.631	90.88
	XGB	82.93	81.97	83.89	0.661	92.02
	LGBM	84.13	82.93	85.33	0.686	93.98
	SVM	78.90	80.12	82.35	0.616	90.22
**CKSAAP**	RF	82.09	82.93	81.25	0.642	86.71
	ETC	83.77	79.8	87.74	0.678	87.02
	XGB	79.44	81.97	76.92	0.592	84.99
	LGBM	83.77	86.05	81.49	0.677	93.01
	SVM	83.81	84.76	81.23	0.662	91.27
**CTDT**	RF	79.44	81.49	77.40	0.590	87.01
	ETC	80.28	81.49	79.08	0.606	89.79
	XGB	78.12	81.73	74.51	0.566	86.04
	LGBM	80.40	82.45	78.36	0.609	90.02
	SVM	75.59	79.95	75.23	0.553	86.42
**CTDC**	RF	82.21	82.93	81.41	0.646	92.71
	ETC	81.97	84.13	79.80	0.641	90.82
	XGB	80.76	81.97	79.56	0.616	89.22
	LGBM	81.49	82.93	80.04	0.631	89.99
	SVM	76.12	79.20	78.11	0.592	86.02
**GDPC**	RF	79.20	82.45	75.96	0.587	87.42
	ETC	80.16	84.12	76.2	0.606	89.12
	XGB	76.92	80.52	73.31	0.541	85.42
	LGBM	78.12	81.97	74.27	0.565	86.13
	SVM	74.12	82.13	75.46	0.524	84.32
**GTPC**	RF	80.88	84.13	77.64	0.621	89.00
	ETC	81.73	80.52	82.93	0.635	89.02
	XGB	77.64	79.08	76.20	0.553	88.52
	LGBM	82.09	83.89	80.28	0.644	86.52
	SVM	75.89	77.02	80.11	0.542	86.52
**DPC**	RF	83.29	82.69	83.41	0.662	85.64
	ETC	82.69	78.60	86.77	0.656	84.23
	XGB	78.00	78.84	77.16	0.561	87.29
	LGBM	81.00	81.25	80.76	0.620	87.29
	SVM	82.20	83.20	82.20	0.646	89.30

Table S3: The performance evaluation of the baseline models on the TF independent set

**Encoding**	**Model**	**ACC**	**SN**	**SP**	**MCC**	**AUC**
**AAC**	RF	80.18	84.90	75.47	0.606	91.22
	ETC	81.60	84.90	78.30	0.633	90.44
	XGB	80.66	85.84	75.47	0.616	86.38
	LGBM	84.44	88.67	80.18	0.691	92.02
	SVM	80.19	89.92	80.01	0.601	89.00
**APAAC**	RF	83.96	83.96	83.96	0.679	90.53
	ETC	82.07	82.07	82.07	0.641	91.26
	XGB	77.35	83.01	71.69	0.550	86.09
	LGBM	85.84	86.79	84.90	0.717	91.27
	SVM	74.53	80.29	78.23	0.540	85.92
**PAAC**	RF	83.01	81.13	84.9	0.66	89.41
	ETC	84.43	84.90	83.96	0.688	90.73
	XGB	79.24	83.01	75.47	0.586	86.68
	LGBM	81.60	85.84	77.35	0.634	89.3
	SVM	76.42	79.90	80.00	0.570	84.09
**CKSAAP**	RF	81.13	84.90	77.35	0.624	90.23
	ETC	83.01	80.18	85.84	0.661	91.37
	XGB	80.18	80.18	80.18	0.603	86.73
	LGBM	79.24	85.84	72.64	0.590	89.52
	SVM	84.91	85.88	96.23	0.669	92.30
**CTDT**	RF	79.71	82.07	77.35	0.595	88.24
	ETC	80.18	83.01	77.35	0.604	88.24
	XGB	76.88	83.96	69.81	0.543	85.98
	LGBM	79.24	83.96	74052	0.587	85.98
	SVM	76.89	80.13	74.83	0.541	84.23
**CTDC**	RF	83.01	85.84	80.18	0.661	89.69
	ETC	80.18	83.01	77.35	0.604	90.23
	XGB	78.77	83.01	74.52	0.577	87.72
	LGBM	81.13	83.96	78.30	0.623	91.45
	SVM	76.89	80.12	79.33	0.562	86.22
**GDPC**	RF	75.00	81.13	68.86	0.503	85.59
	ETC	78.77	81.13	76.41	0.576	87.88
	XGB	69.81	79.44	60.37	0.403	79.44
	LGBM	76.88	84.90	68.86	0.544	86.51
	SVM	72.17	82.12	72.13	0.513	84.52
**GTPC**	RF	80.18	84.9	75.47	0.606	88.29
	ETC	81.13	81.13	81.13	0.622	89.74
	XGB	71.69	74.52	68.86	0.434	82.20
	LGBM	79.71	84.9	74.52	0.5975	88.23
	SVM	76.89	79.04	80.52	0.563	84.23
**DPC**	RF	81.13	81.13	81.13	0.622	88.51
	ETC	82.54	77.35	87.73	0.654	90.58
	XGB	76.41	83.01	69.81	0.532	85.64
	LGBM	78.77	77.35	80.18	0.575	87.29
	SVM	79.25	76.35	84.19	0.569	87.89
